# Accuracy of Algorithms and Visual Inspection for Detection of Trigger Asynchrony in Critical Patients : A Systematic Review

**DOI:** 10.1155/2021/6942497

**Published:** 2021-09-28

**Authors:** Monique Bandeira, Alícia Almeida, Lívia Melo, Pedro Henrique de Moura, Emanuelle Olympia Ribeiro Silva, Jakson Silva, Armèle Dornelas de Andrade, Daniella Brandão, Shirley Campos

**Affiliations:** ^1^Physical Therapy Department, Universidade Federal de Pernambuco, Recife, Brazil; ^2^Hospital Geral Otavio de Freitas, Recife, Brazil

## Abstract

**Objective:**

This study aimed to summarize the accuracy of the different methods for detecting trigger asynchrony at the bedside in mechanically ventilated patients.

**Method:**

A systematic review was conducted from 1990 to 2020 in PubMed, Lilacs, Scopus, and ScienceDirect databases. The reference list of the identified studies, reviews, and meta-analyses was also manually searched for relevant studies. The reference standards were esophageal pressure catheter and/or electrical activity of the diaphragm. Studies were assessed following the QUADAS-2 recommendations, while the review was prepared according to the PRISMA criteria.

**Results:**

One thousand one hundred and eleven studies were selected, and four were eligible for analysis. Esophageal pressure was the predominant reference standard, while visual inspection and algorithms/software comprised index tests. The trigger asynchrony, ineffective expiratory effort, double triggering, and reverse triggering were analyzed. Sensitivity and specificity ranged from 65.2% to 99% and 80% to 100%, respectively. Positive predictive values reached 80.3 to 100%, while the negative predictive values reached 92 to 100%. Accuracy could not be calculated for most studies.

**Conclusion:**

Algorithms/software validated directly or indirectly using reference standards present high sensitivity and specificity, with a diagnostic power similar to visual inspection of experts.

## 1. Introduction

Patient-ventilator asynchrony (PVA) consists of incoordination between the patients ventilatory need and mechanical support [1]. Twenty-five percent of patients under artificial ventilation experience some asynchronous event [2]. Those asynchronies not corrected are associated with abusive use of sedatives, respiratory muscles damage, prolonged time on mechanical ventilation (MV), cognitive changes, sleep disturbance, dynamic hyperinflation, and lung injury [3–5], thus contributing to weaning failure, increased length of stay in the intensive care unit (ICU), and higher mortality risk [6, 7].

Several methods are considered the gold standard to detect PVA, including the esophageal pressure (Pes) analysis [8], the association between Pes and electrical activity of the diaphragm (EAdi), or diaphragmatic neurogram [8–10]. Nevertheless, these methods are commonly applied in clinical research and certain ICU cases. The limitation of use in the daily routine is due to the fact that both are an additional invasive device, and it may also be related to the technical specificities of insertion and proper placement of the catheter, feasibility, and the interpretation of the measurements [3, 8, 11, 12].

Noninvasive methods include graphical analysis of pressure, volume, and flow waveforms at the bedside, system analysis, or software for automatic PVA detection [2, 13]. In this context, software development for reliable and reproducible PVA detection is growing, and there is a great methodological diversity to detect the different types of asynchronies.

Considering that trigger asynchrony is the most frequent type in the ICU and represents at least 80% of asynchronous events [1, 7, 14], the present study proposes a systematic review to summarize the accuracy of methods to diagnose trigger asynchrony at the bedside in patients with respiratory failure using the Pes catheter and the EAdi as reference standards and, therefore, assist intensive care professionals in early diagnosis and clinical management.

## 2. Methods

### 2.1. Review Stage

This systematic review was registered in the International Prospective Register of Systematic Reviews (PROSPERO) database (Register no. CRD42020203676). Accurately, prospective cross-sectional observational, retrospective, and validation studies, involving mechanically ventilated patients of both sexes, aging ≥18 years, and presenting at least two methods for detecting PVA (including a reference standard) were included.

Those studies that did not evaluate the accuracy of methods for detecting PVA or the trigger asynchrony, with patients under noninvasive mechanical ventilation, who were not admitted to the ICU, and/or those mechanically ventilated with sleep disorders or neuromuscular diseases were excluded.

The reference standards were Pes and EAdi. The comparison methods were the visual inspection of pressure, volume, and flow waveforms and software/algorithms for automatic detection. Further analysis between software/algorithms (already validated using a reference standard) and visual inspection were also discussed. The terms concerning trigger asynchronies and the methods for PVA detection are described in Supplementary File 1.

An exhaustive search using indexers combined with the Boolean logic operators “AND,” “OR,” and “NOT” was conducted within the following databases: Medical Literature Analysis and Retrieval System Online (Medline) databases via PubMed (from 1990 to 2020), Latin American and Caribbean Health Sciences Literature (Lilacs) (from 1990 to 2020), SciVerse Scopus (Scopus) (from 1990 to 2020), and ScienceDirect (from to 1990 to 2020 (Supplementary File 2). The keywords and synonyms used were established with no language or publication status restriction and based on the Health Sciences (DeCS) and Medical Subject Headings (MeSH) descriptors. The reference lists of the identified studies were manually searched for potentially relevant studies.

### 2.2. Data Collection and Analysis

Initially, two researchers (MB and AC) independently searched the articles using the predetermined indexers and read the titles and abstracts. Studies that were potentially relevant or raised questions were retained for careful full-text analysis. The articles in which both reviewers agreed to include were retained for further analysis, while disagreements were resolved by a third researcher (SLC). The selection process for this systematic review is presented in a flowchart according to the Preferred Reporting Items for Systematic Reviews and Meta-Analyses (PRISMA) (Supplementary File 3).

### 2.3. Risk of Bias Assessment and Data Synthesis

The characteristics of each study and the risk of bias were performed following the Quality Assessment of Diagnostic Accuracy Studies (QUADAS-2) using the Review Manager 5.4 software (RevMan 5.4, The Nordic Cochrane Centre, Copenhagen, Denmark) (Supplementary File 4).

## 3. Results

The database search generated 1111 results, and after removing 50 duplicates and reading 1061 abstracts, only 49 articles were selected for full-text analysis. Of these, 45 were excluded for being conducted in a noninvasive ventilatory mode, without data regarding the accuracy and for not presenting outcome measures for this study. Therefore, only four of these met the eligibility criteria and were included in this study: Chang et al. [15], Chen et al. [16], Blanch et al. [17], and Rodriguez et al. [18].

### 3.1. Study Characteristics

The included studies were conducted in Taiwan (2007 and 2008) [15], [16], Spain (2012) [17], and Argentina (2019) [18] and used software and visual inspection methods as diagnostic tests (index tests). It can be highlighted that the criteria for defining trigger asynchrony, the algorithms used, and agreement measures among experts were different between studies. The descriptive characteristics and outcomes of interest of the included studies are shown, and the qualitative analysis is shown in Supplementary File 5.

### 3.2. Individual Study Results

The outcomes of interest are shown in Supplementary File 6.

### 3.3. Lan Chang, Pau-Choo Chung, and Chang-Wen Chen, 2007

This study proposed to evaluate the combination of neural network and wavelet feature extraction for trigger asynchrony detection (defined as 1 cmH_2_O Pes drop, drop of airway pressure, and rise of airway flow) in 7 breath sequences, each one lasting more than 1,000 seconds.

A neural network analysis was performed to evaluate the inspiratory and expiratory phases separately. The comparison between the neural network and visual inspection of airway flow and pressure waveforms showed satisfactory accuracy. However, situations with very few trigger asynchronies can generate insufficiency in the true positive statistic. In the future, trigger asynchrony properties, including width, depth, and main frequency band shape, should be explored to refine the algorithm and achieve better results.

### 3.4. Chang-Wen Chen, Wei-Chieh Lin, Chih-Hsin Hsu, Kuo-Sheng Cheng, and Chien-Shun Lo, 2008

In this work, an algorithm was analyzed to detect ineffective triggering in the expiratory phase (ITE) in 24 mechanically ventilated patients with acute respiratory failure. Pes (reference standard for detecting inspiratory effort) and airway pressure and flow waveforms were recorded for a period of 10–30 minutes. Only 14 patients presented ITEs in 1,831 out of the 5,899 breath segments analyzed. Of these, 1,703 had only one asynchronous event, while 128 segments contained multiple ineffective triggering.

Initially, airway flow and pressure waveforms were visually analyzed without visible Pes tracing to verify whether patients presented ITE before proceeding to the analysis using the algorithm. Three ICU specialists, doctors, or respiratory therapists performed visual inspection. The sensitivity and specificity for this method were 93.4% and 96%, respectively.

The distribution of flow and pressure waveform deflection was performed to quantify the diagnostic performance of these two variables when determining the ITE using the algorithm. The optimal values adopted for detecting the asynchronous events were 5.45 L/min for maximum flow detection (sensitivity and specificity values of 91.5% and 96.2%, respectively) and 0.45 cmH_2_O for maximum airway pressure deflection (sensitivity of 93.3% and specificity of 92.9%).

By analyzing the segments containing multiple ITEs, a higher maximum airway pressure deflection was found. Also, these expiratory segments lasted more than 2.95 seconds. Therefore, ITEs detection was performed with an algorithm based only on a maximum airway pressure deflection cutoff value of 0.74 cmH_2_O and an expiratory segment length of 2.95 seconds, resulting in a sensitivity of 88.4% and a specificity of 98.8%.

Therefore, this algorithm presented good accuracy, and its sensitivity and specificity values were comparable with visual inspection of airway flow and pressure waveforms.

### 3.5. Blanch et al., 2012

Blanch et al. [17] performed a pilot study to determine the accuracy of a computerized system (Better Care®) in automatically identifying ineffective efforts during expiration (IEE) by analyzing flow waveforms. This software estimates the optimal expiratory flow curves, compares with the actual flow of the patient, and expresses this difference as percentage value. An ineffective expiratory effort was recognized when the actual flow waveform deviated 42% from the ideal expiratory curve. This method was compared with visual inspection (five independent specialists in this area) and EADi (neurally adjusted ventilatory catheter).

The total study sample was subdivided into two groups of eight patients. The first group compared the Better Care® software and the visual inspection method using data from 1,024 randomly selected breaths, and the following results were observed: a sensitivity of 91.5%, specificity of 91.7%, positive predictive value of 80.3%, negative predictive value of 96.7%, and kappa index of 79.7% (95% confidence interval (CI): 75.6% to 83.8%). In the second group, the algorithm was validated using EADi with data from 9,600 breaths. Compared with EADi, the IEE algorithm presented a sensitivity of 65.2%, specificity of 99.3%, positive predictive value of 90.8%, negative predictive value of 96.5%, and kappa index of 73.9% (95%CI: 71.3% to 76.3%).

Therefore, the Better Care® software was able to identify IEE during MV assistance with similar precision to the visual inspection method and EADi.

### 3.6. Rodriguez et al., 2019

Rodriguez et al. [18] developed an algorithm based on airway flow and pressure signals to classify breaths as normal, reverse triggering asynchrony (RT) with or without breath-stacking (BS), and patient-initiated double triggering (DT). An esophageal balloon was used as the reference standard. Therefore, this study aimed to validate an algorithm to detect these changes in patient-ventilator interaction.

The diagnostic performance of the algorithm was validated using two classifications. The first was based on visual inspection of the Pes signal of 699 breaths recorded in 11 patients with acute respiratory distress syndrome. The other was obtained by visual inspection (2 physicians and 5 physiotherapists) of pressure and airway flow signals of 1881 breaths (99 patients). The RT with or without BS represented 19% and 37% of breaths in the Pes dataset, while their frequency in the specialists' dataset was 3% and 12%, respectively. The DT was very rare in both datasets. The algorithm classification accuracy was 0.92 (95% CI: 0.89–0.94, *P* < 0.001) and 0.96 (95% CI: 0.95–0.97, *P* < 0.001) compared with Pes and visual inspection (kappa values were 0.86 and 0.84, respectively). The algorithm precision, sensitivity, and specificity for individual asynchronies were excellent. This algorithm yields an excellent precision to detect clinically relevant asynchronies related to RT.

## 4. Clinical Impact

Asynchrony detection using algorithm/software demonstrated sensitivity and specificity values similar to expert visual inspection in all included studies.

Despite differences between the standard references, all studies presented specificity values higher than 90%. The algorithm proposed by Chen et al. [16] presented excellent sensitivity and specificity values for maximum flow deflection and maximum pressure deflection, and results were comparable with visual inspection. Although Rodriguez et al. presented excellent sensitivity and specificity values for RT without BS, the algorithm was more sensitive and specific for detecting RT with BS in both analyses (esophageal pressure and visual inspection).

The software proposed by Blanch et al. presented excellent sensitivity and specificity values compared with visual inspection by ICU professionals, a finding similar to the studies of Chen et al. [16], Rodriguez et al. [18], and Chang et al. [15]. However, the comparison between the algorithm and EADi showed a drop in sensitivity and high specificity to detect the triggering asynchronies. This study was limited to patients without phrenic nerve injury and neuromuscular disease since these conditions could interfere with EADi.

Some factors may influence signal reliability when creating algorithms based on Pes and EAdi signals, such as retained airway secretion, cardiac oscillations, and expiratory muscle contraction and relaxation [16]. In the study by Chen et al. [16], tracheal aspiration was performed before data collection; therefore, the noise was not filtered, reducing the clinical applicability since airway secretion is common in the ICU environment. In the study by Blanch et al., the software was built without controlling airway secretions; however, this instrument was closer to clinical reality due to its good sensitivity and specificity.

Despite the evolution, the mechanical ventilators are still unable to automatically detect PVA [1, 17]. The automatic detection of trigger asynchrony using software/algorithms presented sensitivity and specificity values comparable to visual inspection of flow and pressure waveforms in all included studies.

A desirable solution is that mechanical ventilators become increasingly autonomous, responsive and intelligent, capable of delivering continuously adjusted ventilation for monitoring respiratory parameters or detected needs, making it more comfortable and optimized [12, 19].

The efforts that have taken place to detect and classify asynchronies, such as those described in this review, have already stimulated the development, use, and incorporation of algorithms, software or tools in some commercially available mechanical ventilators for a finer control of ventilatory synchronization, such as the Better Care® system, IntelliSync + technology on the Hamilton Medical ventilators, Dragger's Primus® software, Puritan Bennett™ 980 Leak Sync Software, and the Bellavista™ 1000 ventilator.

The visual inspection is an alternative for automatic detection by software and algorithms since it is a simple and low-cost method. Furthermore, professional training and capacity building are essential for identifying asynchronous events using graphical monitoring since availability and applicability of more invasive, high-cost, or sophisticated methods are limited, and PVA may lead to increased MV duration, length of ICU stay, number of tracheostomies, and hospital costs [1, 6].

A significant limitation is present in the applicability of evidence. In general, the studies were heterogeneous regarding the patient number, reference standard methods, diseases, and MV brands (which compromises different flow and pressure curve analyses), ventilatory modes, and parameters. Therefore, the meta-analysis was not performed in this review.

Further research regarding methods for detecting asynchrony should take into account sample size, patient selection, protocol standardization, ventilatory modes and parameters, and other types of asynchronies.

## 5. Conclusions

Algorithms/software designed for the automatic detection of trigger asynchrony using Pes and EADi as reference standard present high sensitivity and specificity; however, they were similar to expert visual inspection. Further studies are necessary to increase the accuracy of these methods at the bedside and apply to different situations.

## Data Availability

The data supporting this SYSTEMATIC REVIEW are from previously reported studies and datasets, which have been cited. The processed data are available in the article.

## Additional Points

*Clinical Significance.* Algorithms/software for automatic detection of trigger asynchrony are a promising innovation for the ventilatory management, with potential impact in reducing errors, more safety, and efficiency in care of critical patients.

## Supplementary Materials

Supplementary Materials Supplementary File 1. Definition of terms regarding trigger asynchrony and detection methods. Supplementary File 2. Search strategy. Supplementary File 3. Flowchart of the article selection process according to the Preferred Reporting Items for Systematic Reviews and Meta-Analyses (PRISMA). Supplementary File 4. Risk of bias analysis. Supplementary File 5. Risk of bias in individual studies. Supplementary File 6. Study results.
