# Supplementary material for: Accuracy of Algorithms and Visual Inspection for Detection of Trigger Asynchrony in Critical Patients : A Systematic Review
Source: Crit Care Res Pract. 2021 Sep 28;2021:6942497. doi: 10.1155/2021/6942497 (PMC8492248; doi:10.1155/2021/6942497)
Supplement: Supplementary Materials — Supplementary File 1. Definition of terms regarding trigger asynchrony and detection methods. Supplementary File 2. Search strategy. Supplementary File 3. Flowchart of the article selection process according to the Preferred Reporting Items for Systematic Reviews and Meta-Analyses (PRISMA). Supplementary File 4. Risk of bias analysis. Supplementary File 5. Risk of bias in individual studies. Supplementary File 6. Study results. [file 6942497.f1.docx]

**SUPPLEMENTARY FILE 1**

**Definition of terms regarding trigger asynchrony and detection methods**

**Patient-ventilator asynchrony**

It is the imbalance between the patient's ventilatory demand and the mechanical ventilator adjustments. The patient presents different inspiratory and expiratory times than those offered by the ventilator [1].

**Defining trigger asynchronies**

Ineffective triggering

When the inspiratory effort is not enough to open the inspiratory valve of the mechanical ventilator. The airway and esophageal pressures drop, while expiratory flow raises; however, the effort is not enough to trigger the ventilator [2] - [3].

Self-triggering

It occurs when the ventilatory cycle is triggered without an effective patient effort. It may occur due to circuit leakage/condensation, heart rate detection, and large variations in intrathoracic pressure [2] - [3].

Double-triggering

It occurs when two ventilatory cycles are triggered after a single inspiratory effort. It is usually due to high ventilatory demands, leading to shorter inspiratory and expiratory times between cycles [2] - [3].

Reverse triggering

It occurs when mechanical insufflation triggers respiratory muscle contraction. The inspiratory time between cycles is reduced or does not occur compared with normal cycles [4].

**Methods for detecting patient-ventilator interaction**

Esophageal or transdiaphragmatic pressure

It allows the direct monitoring of diaphragm activity by inserting an esophageal balloon catheter. A “gold standard” diaphragmatic contraction can be monitored, and specific alterations can be diagnosed [4].

Electrical activity of the diaphragm (EAdi)

It is obtained using a catheter comprised of electrical sensors that capture diaphragmatic contraction and allows continuous and accurate monitoring of diaphragmatic behavior, therefore, enabling synchrony between the adjusted parameters of the mechanical ventilator and the respiratory demand of the patient [5] - [6].

Software/Algorithm

This method is used in many studies. It comprises the analysis of online and offline graphs for short periods; however, the commercial interest generated improvements and currently is an effective resource for continuous monitoring. Some of these softwares can detect asynchronies automatically or using standard methods [7].

Mechanical ventilator waveforms

Are presented on the mechanical ventilator monitor and represent volume, pressure, and flow waveforms that partially correlate with diaphragmatic activity. Limitations are related to the professional's ability to detect and interpret the signal changes, the lack of sound or visual alerts, and the poor detection of some specific asynchronies using only these signals [8].

References

[1] A. W. Thille, P. Rodriguez, B. Cabello, F. Lellouche, and L. Brochard, “Patient-ventilator asynchrony during assisted mechanical ventilation”, *Intensive care medicine*, vol. 32, n^o^ 10, p. 1515–1522, 2006.

[2] S. C. Publio and J. A. Martins, “Interação paciente ventilador nas diferentes fases do ciclo ventilatório em ventilação por pressão de suporte”, *Rev Med Minas Gerais*, vol. 20, n^o^ 3 Supl 4, p. S55–S65, 2010.

[3] M. De Wit, K. B. Miller, D. A. Green, H. E. Ostman, C. Gennings, and S. K. Epstein, “Ineffective triggering predicts increased duration of mechanical ventilation”, *Critical care medicine*, vol. 37, n^o^ 10, p. 2740–2745, 2009.

[4] E. Akoumianaki *et al.*, “The application of esophageal pressure measurement in patients with respiratory failure”, *American journal of respiratory and critical care medicine*, vol. 189, n^o^ 5, p. 520–531, 2014.

[5] T. Mauri et al. ,“Patient-ventilator interaction in ARDS patients with extremely low compliance undergoing ECMO: A novel approach based on diaphragm electrical activity”. *Intensive Care Medicine*, v. 39, n. 2, p. 282–291, 2013.

[6] L.Piquilloud et al. , “Neurally adjusted ventilatory assist (NAVA) improves patient-ventilator interaction during non-invasive ventilation delivered by face mask”. *Intensive Care Medicine*, v. 38, n. 10, p. 1624–1631, 2012.

[7] L. Blanch *et al.*, “Validation of the Better Care® system to detect ineffective efforts during expiration in mechanically ventilated patients: a pilot study”, *Intensive care medicine*, vol. 38, n^o^ 5, p. 772–780, 2012.

[8] D. Colombo *et al.*, “Efficacy of ventilator waveforms observation in detecting patient–ventilator asynchrony”, *Critical care medicine*, vol. 39, n^o^ 11, p. 2452–2457, 2011.

**SUPPLEMENTARY FILE 2**

**Search strategy**

| Search strategy |  |
| --- | --- |
| **Database** |  |
| Lilacs | (tw:(patient ventilator asynchrony) AND (tw:(“visual inspection” OR “waveform analysis” AND (tw:(mechanical ventilation)) in Title, Abstract or Keyword and (((diaphragmatic electrical activity) AND (mechanical ventilation)) AND (synchrony)) AND (adults) in Title, Abstract or Keyword and (tw:(transdiaphragmatic) AND (pressure)) AND (mechanical ventilation)) AND (asynchronies) in Title, Abstract or Keyword and (tw:((asynchrony) AND (mechanical)) AND (ventilation)) AND (detection) |
| PubMed | ((patient ventilator asynchrony) AND (visual inspection)) AND (mechanical ventilation) ((patient ventilator asynchrony) AND (waveform analysis)) AND (mechanical ventilation) (((asynchrony) AND (mechanical)) AND (ventilation)) AND (detection) (((diaphragmatic electrical activity) AND (mechanical ventilation)) AND (synchrony)) AND (adults) (((transdiaphragmatic) AND (pressure)) AND (mechanical ventilation)) AND (asynchronies) |
| ScienceDirect | (tw:(patient ventilator asynchrony)) AND (tw:(visual inspection)) AND (tw:(mechanical ventilation)) in Title, Abstract or Keyword and ((patient ventilator asynchrony)) AND (tw:(waveform analysis)) AND (tw:(mechanical ventilation)) in Title, Abstract or Keyword and (((asynchrony) AND (mechanical)) AND (ventilation)) AND (detection) in Title, Abstract or Keyword and (tw:(diaphragmatic electrical activity)) AND (tw:(mechanical ventilation)) AND (tw:(synchrony)) AND (tw:(adults)) in Title, Abstract or Keyword and (tw:(transdiaphragmatic)) AND (tw:(pressure)) AND (tw:(mechanical ventilation)) AND (tw:(asynchronies)) in Title, Abstract or Keyword |
| Scopus | (TITLE-ABS-KEY(“asynchrony” OR ”synchrony” OR “assynchronies”) AND (TITLE-ABS-KEY (mechanical) AND (TITLE-ABS-KEY(ventilation) AND (TITLE-ABS-KEY(detection) (TITLE-ABS-KEY(patient ventilator asynchrony) AND (TITLE-ABS-KEY(“waveform analysis” OR “visual inspection”) AND (TITLE-ABS-KEY(mechanical ventilation) AND (TITLE-ABS-KEY(diaphragmatic electrical activity) AND (TITLE-ABS-KEY(mechanical ventilation) AND (TITLE-ABS-KEY(adults) (TITLE-ABS-KEY(transdiaphragmatic pressure)) |
|  |  |

**SUPPLEMENTARY FILE 3**

**Flowchart of the article selection process according to the Preferred Reporting Items for Systematic Reviews and Meta-Analyzes (PRISMA).**

Additional records identified through other sources
(n = 03)

Records identified through database searching
(n = 1108)

## Identification

Records after duplicates removed
(n = 106)

## Screening

Records excluded after abstracts screening
(n = 1012)

Records screened
(n =1061)

Full-text articles assessed for eligibility
(n = 4)

Full-text articles excluded, with reasons
(n = 45)

## Eligibility

Studies included in qualitative synthesis
(n = 4)

## Included

**SUPPLEMENTARY FILE 4**

**Risk of bias analysis**

**Risk of bias**

For the risk of bias analysis the studies were divided according to the reference standard:

- Esophageal pressure [1]-[2]-[3]

Low risk of bias in all analyzed domains, except for the study by Chang et al., who presented a high risk of bias in three domains.


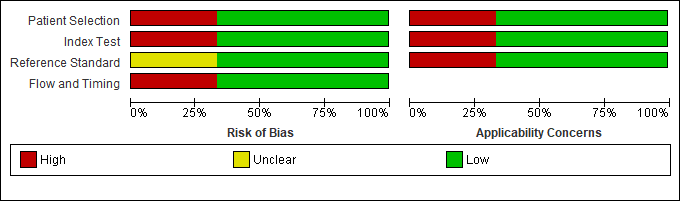


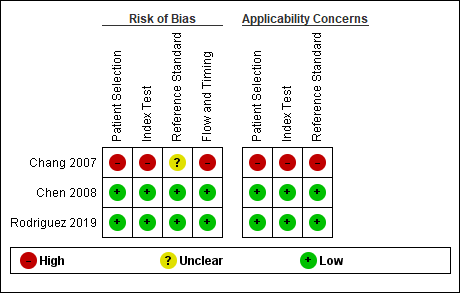


- The studies by Lan Chang, Pau-Choo Chung and Chang-Wen Chen [1], Chang-Wen Chen, Wei-Chieh Lin, Chih-Hsin Hsu, Kuo-Sheng Cheng and Chien-Shun Lo [2], and Rodriguez et al. [3] were performed using an esophageal pressure catheter as a reference standard. Although Chen et al. and Rodriguez et al. did not meet all the risk of bias criteria, the study designs were relatively homogeneous and presented a low risk of bias in the four domains analyzed (Appendix 5). However, Chang et al. presented an uncertain risk of bias for the reference standard and a high risk of bias in the following domains: patient selection, index test, flow, and timing.
- The algorithm proposed by Chang-Wen Chen, Wei-Chieh Lin, Chih-Hsin Hsu, Kuo-Sheng Cheng and Chien-Shun Lo [2] presented excellent sensitivity and specificity values for the maximum flow and pressure deflections and was comparable to visual detection. The same could be observed in the studies by Rodriguez et al. [3] and Chang et al. [1]. Although Rodriguez et al. presented excellent sensitivity and specificity values for reverse-triggering without breath-stacking, the algorithm was more sensitive and specific for detecting reverse-triggering with breath-stacking in both analyzes (with esophageal pressure and visual inspection).
- Electrical activity of the diaphragm (EAdi) [4]

It presented a low risk of bias in all domains, except in the domain regarding flow and timing.


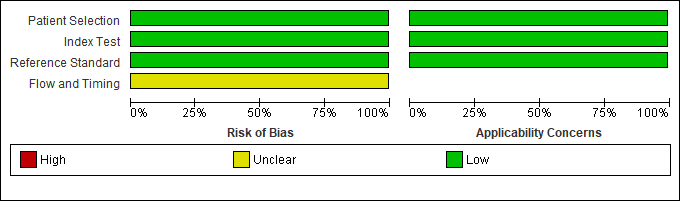


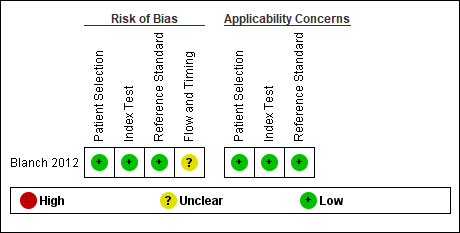


The study by Blanch et al. [4] used EADi through a NAVA catheter as a reference standard. It also did not meet all the risk of bias criteria, demonstrating uncertain risk regarding the second item of domain 3 and the first item of domain 4. However, study design was very homogeneous and a low risk of bias was observed in all domains analyzed, except for domain 4 (uncertain risk of bias) (Supplementary File 5).

References

[1] L. Chang, P.-C. Chung, and C.-W. Chen, “Combining Neural Network and Wavelet Transformn for Trigger Asynchrony Detection”, 2007.

[2] C.-W. Chen, W.-C. Lin, C.-H. Hsu, K.-S. Cheng, and C.-S. Lo, “Detecting ineffective triggering in the expiratory phase in mechanically ventilated patients based on airway flow and pressure deflection: feasibility of using a computer algorithm”, *Critical care medicine*, vol. 36, n^o^ 2, p. 455–461, 2008.

[3] P. O. Rodriguez *et al.*, “Automatic detection of reverse-triggering related asynchronies during mechanical ventilation in ARDS patients using flow and pressure signals”, *Journal of Clinical Monitoring and Computing*, p. 1–8, 2019.

[4] L. Blanch *et al.*, “Validation of the Better Care® system to detect ineffective efforts during expiration in mechanically ventilated patients: a pilot study”, *Intensive care medicine*, vol. 38, n^o^ 5, p. 772–780, 2012.

**SUPPLEMENTARY FILE 5**

**Risk of bias in individual studies**

|  |  |  |  | | |
| --- | --- | --- | --- | --- | --- |
|  | **Item** | **EADi *versus* Algorithm (validated using the Pes versus VI)** | **Pes *versus* Algorithm (validated using the Pes versus VI)** | | |
|  |  | **Blanch et al., 2012** | **Chang et al., 2007** | **Chen et al., 2008** | **Rodriguez et al., 2019** |
| **Domain 1:**  Patient selection | Consecutive or random sampling? | Y | U | Y | Y |
|  | Avoided case-control design? | Y | U | Y | Y |
|  | Avoided inappropriate exclusions? | Y | U | Y | U |
|  | **Patient selection introduced bias?** | L | H | L | L |
|  | **Applicability issues**: Was there a concern that patients and the included configurations did not correspond to the review objectives? | L | H | L | L |
| **Domain 2:** Diagnostic test (Index Test) | Were the diagnostic test (index test) results interpreted without observing the results of the reference standard? | Y | U | U | U |
|  | If a threshold was used, was it pre-specified? | Y | Y | Y | Y |
|  | Did the performance or interpretation of the diagnostic test (index test) introduce bias? | L | H | L | L |
|  | **Applicability question**: Did the diagnostic test (index test) or its conduction/interpretation differed from the review objectives? | L | H | L | L |
| **Domain 3:** Reference standard | Did the reference standard classify the condition correctly? | Y | Y | Y | Y |
|  | Were the reference standard results interpreted without knowing the results of the diagnostic test (index test)? | U | U | U | U |
|  | **Did the reference standard and its conduction/interpretation introduce bias?** | L | U | L | L |
|  | **Applicability issues:** Were there concerns that the condition, defined by the reference standard, did not match the review objectives? | L | H | L | L |
| **Domain 4:** Flow and time | Was there an appropriate interval between the diagnostic test (s) (index tests) and the reference standard? | U | U | U | U |
|  | Did all patients receive the same reference standard? | Y | Y | Y | Y |
|  | Were all patients included in the analysis? | Y | Y | Y | Y |
|  | **Did the patient flow introduce bias?** | U | H | L | L |
|  |  |  |  |  |  |

EAdi: Electrical activity of the diaphragmatic. VI: Visual inspection. Pes: Esophageal pressure. N, no; Y, yes; U, uncertain. Risk/Concern: H, high; L, low.

| SUPPLEMENTARY FILE 6  Study Results | | | | | | |
| --- | --- | --- | --- | --- | --- | --- |
| Author/year/  country | **Reference standard** | **Criteria index** | **Analysis** | **Results** | | |
|  |  |  |  | **Sensitivity/**  **Specificity** | **PPV/NPV** | **Additional results** |
| Chang et al. 2007 Taiwan | Pes. | Visual inspection: Airway pressure and flow | Algorithm versus doctor or therapist. | - | - | CSCR: 100.75%; TPCR: 72.68%, TAMCR: 407.10% |
| Chen et al. 2008  Taiwan | Pes. | Fdef calculated using the algorithm | Algorithm validated using Pes and Fdef for all ITEs | 91.5%/96.2% | - |  |
|  |  | Pdef calculated using the algorithm | Algorithm validated using Pes and Fdef for all ITEs | 93.3% /92.9% | - |  |
|  |  |  | Algorithm validated using Pes and Fdef for multiple ITEs | 88.4%/ 98.8% |  |  |
|  |  | Visual inspection: Airway pressure and flow | ITE visual inspection without visible Pes tracings | 93.4%/96% | - |  |
| Blanch et al. 2012 Spain | EAdi | IEE cutoff point determined based on the experts’ analyses. | IEE analysis by expert ICU professionals and Better Care^®^ software | 65.2%/99.3% | 90.8%/96.5% | Kappa coefficient = 73.7%  AUC= 0.964 (95%CI = 0.952 - 0.975) |
|  |  | Expiratory airflow waveforms | EADi signal *versus* Better Care^®^ algorithm | 91.5%/91.7% | 80.3%/96.7% | Kappa coefficient = 79.7% |
| Rodriguez et al. 2019  Argentina | Pes. | Algorithm | Algorithm *versus* Pes normal classification | 97%/91% | 89%/97% | Kappa coefficient = 0.86 (general)  Accuracy = 0.92 |
|  |  |  | Algorithm  *versus* Pes classification: RT without BS | 86%/98% | 96%/92% |  |
|  |  |  | Algorithm *versus* Pes classification: RT with BS | 90%/100% | 100%/98% |  |
|  |  | Visual inspection: Airway pressure and flow | Algorithm *versus* experts’ classification | 99%/80% | 96%/95% | Accuracy: 0.96  Kappa coefficient: 0.84 |
|  |  |  | Algorithm *versus* experts’ classification: RT without BS | 74%/99% | 94%/97% |  |
|  |  |  | Algorithm *versus* experts’ classification: RT with BS | 89%/100% | 93%/100% |  |

Pes: Esophageal pressure; EADi: Electrical activity of the diaphragm; CSCR: Correct segments comparing ratio; TPCR: True positive rate; TAMCR: Trigger asynchrony misrecognition comparing ratio; ITE: Ineffective triggering in the expiratory phase; Pdef: Maximum airway pressure deflection; Fdef: Maximum flow detection; ICU: Intensive care unit; PPV: Positive predictive value; NPV: Negative predictive value: AUC: Area under the curve; CI: Confidence interval; IEE: Ineffective efforts during expiration; RT: Reverse triggering; BS: Breath-stacking
